# THE ASSOCIATION BETWEEN GRATITUDE AND MORTALITY AMONG OLDER US WOMEN

**DOI:** 10.1093/geroni/igae098.2244

**Published:** 2024-12-31

**Authors:** Ying Chen, Olivia Okereke, Eric Kim, Henning Tiemeier, Laura Kubzansky, Tyler VanderWeele

**Affiliations:** Harvard T. H. Chan School of Public Health, Cambridge, Massachusetts, United States; Massachusetts General Hospital, Boston, Massachusetts, United States; University of British Columbia, Vancouver, British Columbia, Canada; Harvard T. H. Chan School of Public Health, Boston, Massachusetts, United States; Harvard T. H. Chan School of Public Health, Boston, Massachusetts, United States; Harvard T. H. Chan School of Public Health, Boston, Massachusetts, United States

## Abstract

Gratitude, as a positive emotion, is a potentially modifiable psychological factor that may enhance healthy aging. However, the association between gratitude and mortality has not been studied. Using data from the Nurses’ Health Study (N=52,169 U.S. female nurses, mean age=79 years, 2016 questionnaire wave to December 2019), Cox proportional hazards regression models estimated the hazard ratio of deaths by self-reported levels of gratitude at baseline. Gratitude was assessed with the 6-item Gratitude Questionnaire, a validated measure of one’s general tendency to experience grateful affect. Deaths were identified from the National Death Index, state statistics records, reports by next of kin, and the postal system. Causes of death were ascertained by physicians through reviewing death certificates and medical records. Over 158,374 person-years of follow-up, 5,343 incident deaths were identified. Greater gratitude was associated with a substantially lower hazard of mortality. For instance, the highest vs. lowest tertile of gratitude was associated with a 10% lower hazard of all-cause deaths (95% confidence interval of hazard ratio: 0.82, 0.97), adjusting for baseline sociodemographic characteristics, social participation, religious involvement, physical health, lifestyle factors, and mental health. When considering cause-specific deaths, gratitude was inversely associated with each specific cause of death. This inverse association was strongest with deaths due to cardiovascular disease. This study provides the first empirical evidence suggesting that experiencing grateful affect is associated with a lower risk of mortality in later life. Interventions designed to increase the experience of grateful affect may provide a novel avenue for increasing longevity among older adults.

